# Brief fruit and vegetable messages integrated within a community physical activity program successfully change behaviour

**DOI:** 10.1186/1479-5868-4-12

**Published:** 2007-04-10

**Authors:** Shawna E Doerksen, Paul A Estabrooks

**Affiliations:** 1 Department of Kinesiology, Kansas State University, Manhattan, KS, USA; 2 Clincal Research Unit, Kaiser Permanente Colorado, Denver, CO, USA; 3 University of Illinois at Urbana-Champaign, USA

## Abstract

**Background:**

Consumption of the recommended amounts of fruits and vegetables is associated with several health benefits. Currently less than 25% of the American population meets the minimum recommendation of five servings a day. In order to change this health behaviour, interventions should be based on theory and include community-wide social support.

**Methods:**

A low intensity intervention was developed in which participants (n = 86) were randomly assigned to either the fruit and vegetable intervention (FVI) or standard control condition. The intervention was integrated into an ongoing community physical activity program and study participants were drawn from the sample of community members enrolled in the program. The FVI consisted of brief social cognitive theory-based messages delivered in nine weekly newsletters designed to improve participant outcome and self-efficacy expectations related to fruit and vegetable consumption.

**Results:**

Participants in the FVI condition increased in their fruit and vegetable consumption by approximately one to one and one-third servings per day. The control condition showed no change in consumption. The effect of the intervention was enhanced when examined by the extent to which it was adopted by participants (i.e., the number of newsletters read). Those participants who read seven or more newsletters showed an increase of two servings per day.

**Conclusion:**

This intervention was effective at improving fruit and vegetable consumption among adults. Minimal interventions, such as newsletters, have the ability to reach large audiences and can be integrated into ongoing health promotion programs. As such, they have potential for a strong public health impact.

## Background

Eating an adequate amount of fruits and vegetables (F&V) provides essential nutrients for growth and maintenance of healthy tissues, bolsters immune function, and is protective against chronic disease [1-7]. To realize these health benefits consuming between five and nine servings per day is recommended [8]. Based on the most recent Behavioral Risk Factor Surveillance Survey, fewer than one in four adults consume five or more servings of F&V. In Kansas where the present investigation was conducted, this rate is closer to one in five [9]. Further, sparsely populated rural areas are often at a disadvantage since they have limited access to food stores and F&V tend to be more expensive than in metropolitan areas [10]. From these data, it is clear that there is a need for behaviour change interventions that target F&V consumption.

The United States Preventive Services Task Force has identified and strongly recommended a number of strategies that have demonstrated effective change across a variety of health behaviours [11-13]. Effective strategies include, among others, community-wide, social support, and theory-based interventions.

However, there is little confirmation that evidence-based health behaviour interventions are routinely being translated into regular practice [14,15]. Glasgow and Toobert [16] suggested that translation efforts could be improved by developing and packaging interventions in a way that is practical, allows for consistent delivery, does not require a great amount of staff or volunteer time to deliver, and can reach and appeal to a high percentage of the target population and practitioners who will ultimately deliver these interventions [16].

In addition Rogers [17] highlighted a number of issues that, when addressed, should improve the likelihood of rapid diffusion of interventions. First, an intervention that is compatible with the mission, goals, and structure of a diffusion system is more likely to be adopted and implemented by that organization. Second, interventions that are complex and difficult to implement are less likely to be adopted and used in a practice setting. Third, an intervention that demonstrates relative advantage over and above the current standard of care will be adopted and implemented at a higher rate than an intervention that is simply compared to a waitlist control or no intervention control group [17].

The general purpose of this study was to develop a behavioural F&V intervention with a high potential for translation into regular practice by; (a) partnering with a delivery system that has a mission to improve public health, (b) developing a parsimonious intervention that is based in sound theory, yet is easy to implement, and (c) demonstrating the effectiveness of the intervention for increasing F&V consumption when compared to the current standard of care.

To achieve this purpose we partnered with the Kansas State University Research and Extension System. The Cooperative Extension System associated with Land Grant Institutions provides an excellent model for the integration of research and practice professionals [18]. Cooperative Extension is a statewide diffusion system that provides an infrastructure with the potential to adopt and deliver health promotion interventions that have a large reach into the population. The primary mission of the Cooperative Extension System is to disseminate and encourage the application of research-based information and programs to individuals, families, and communities. As it is available in every state, and typically has a representative for every county, one of the major advantages of the Cooperative Extension System is its enormous reach into the American population [19].

The Cooperative Extension system associated with Kansas State University has delivered, since the spring of 2002, 'Walk Kansas!' an annual and effective 8-week physical activity promotion program that includes weekly participant newsletters and team-based goal setting [20,21]. This program provides an opportunity to deliver an F&V intervention that could be integrated seamlessly within the newsletter component of the 'Walk Kansas!' program. As such, delivery of the intervention would be easy to implement, not entail increased staff time for delivery, and have a sustainable channel of delivery over time. In addition, the current program delivery would allow for comparison of the F&V intervention to the standard care Walk Kansas newsletters which contain general nutrition information.

## Methods

### Design of study

The present study was a randomized controlled trial that involved volunteer participants already registered in a community-based physical activity program. A blind randomization procedure was used to assign volunteers to either the F&V intervention (FVI) or to a standard-care control condition before the start of the 8-week physical activity program. Data were collected at two time points, once prior to the intervention and again after the completion of the program. Written informed consent was obtained from each participant prior to baseline testing. Study protocol was approved by the Internal Review Board (IRB) at Kansas State University (IRB#2733).

### Selection of subjects

The target population for this study was adults between 18 and 65 years of age who were enrolled in a community-based physical activity promotion program. The sample size required to observe a moderate effect at a power of .80 and probability of .05 was 40 individuals per condition, for a total of 80 participants [22]. Due to the short time frame of the study, a conservative 20% attrition rate was assumed resulting in a target of 50 participants per condition [14].

Participants were recruited from the group of adults participating in a single county offering Walk Kansas (n = 586). All Walk Kansas participants completed a waiver indicating that they were healthy adults without contraindications for regular physical activity. An additional exclusion criterion was developed as part of a larger study not reported in this paper as it does not focus on behaviour change, but rather physiological mechanisms of antioxidants. Exclusion was based upon participant responses to the physical activity questions from the Behavioral Risk Factor Surveillance Survey [23]. Participant responses were analyzed for level of activity. Based on the requirements of the larger study, participants were excluded if they were considered active by CDC/ACSM guidelines [24].

Potential participants were recruited via the telephone, given a detailed description of the study, and asked to participate. If individuals agreed, a time was set up for them to come into the research facilities for testing.

**Figure 1:**
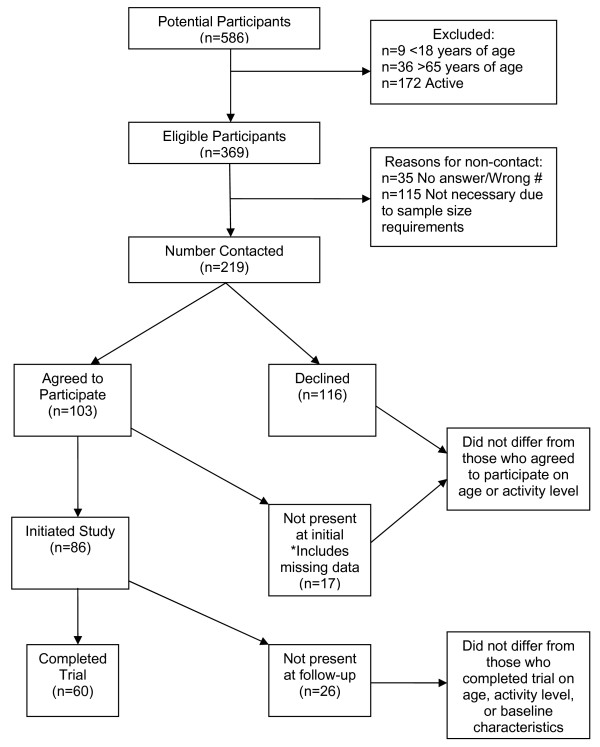
Participant eligibility and recruitment.

Figure 1 shows the flow for subject participation throughout the 8-week study. As can be seen in the figure 63% of the Walk Kansas participants were eligible for the study. Further, 59% of the eligible participants were contacted for possible participation in the study. Finally, of those that were contacted for participation, 47% agreed to participate but only 39% of those contacted completed baseline assessments and began the study.

### Intervention conditions

#### Standard care control condition

Participants assigned to this condition received the entire standard Walk Kansas program. This program was an 8-week, research-based physical activity promotion program utilizing team-building with supportive groups to help participants increase their regular moderate intensity physical activity. Participants joined the program in self-selected teams of six members. Each team identified a group goal of "walking" the equivalent of 423 miles (the width of Kansas). "Walking" was operationalized as any moderate (or higher) intensity physical activity that was done for 15 minutes or more. Distances were recorded as miles. Whereas walking, jogging, and running were documented as actual mileage; all other activities were documented as 15 minutes being equated to one mile. Teams of six were selected because to achieve the 423-mile goal, each participant would need to complete 30 minutes of moderate intensity physical activity a day, five days per week (i.e., the recommended guidelines for regular physical activity).

To enhance motivation, the program also includes nine weekly newsletters. Fundamental to the newsletters are timely updates on each team's mileage and messages based in social cognitive theory to increase and maintain regular physical activity. Additionally, the weekly letters also provided generic nutrition information and healthy recipes. However, these newsletters did not directly address fruit and vegetable consumption.

#### Fruit & Vegetable Intervention (FVI)

This intervention was based on Bandura's [25,26] Social Cognitive Theory (SCT) because of the consistent relationship between self efficacy and eating behaviours [27-30]. The primary personal constructs of SCT include self-efficacy (SE) and outcome expectations (OE). SE is defined as the belief "in one's capabilities to organize and execute the courses of action required to produce given attainments" [26]. OE are defined as the beliefs about the consequences of an action [26]. In the current study, participants in FVI received the same Walk Kansas program as the standard care control group. However, the intervention newsletter included a social-cognitive theory-based message about F&V consumption-'The 5-A-Day Corner' which was prominently displayed on the front page of each of the nine newsletters. The messages (approximately 500-words each) included strategies to increase SE and OE regarding F&V consumption by providing information on such items as preparation techniques and description of the protective effects of F&V. The messages were sequentially designed to enhance OE and provide detailed activities to enhance SE (See Table 1).

**Table 1:** Content of social cognitive messages across weekly newsletters

Week	Social Cognitive Messages
1	Benefits of eating the recommended number of fruits and vegetables
2	Risks of eating too few fruits and vegetables, understanding portion sizes, and accumulating the amount of fruits and vegetables eaten in a day.
3	Strategies to incorporate F&V into main dishes, desserts, breakfast, or an evening snack Tips on planning to have accessible fruits and vegetables in the home.
4	Benefits of eating a variety of fruits and vegetables. Strategies related to using frozen or canned fruits and vegetables and the strategic use of 100% fruit juice
5	Issues of nutritional value related to eating fruits and vegetables raw, cooked, or canned. Tips on preparation to increase the nutritional value of fruits and vegetables.
6	Strategies to prepare fruits and vegetables so that they are more appealing.
7	Strategies to prepare and have fruits and vegetables available so they fit in the daily routine.
8	Top 12 List on how to get 5 a Day; numbers 7–12.
9	Top 12 List on how to get 5 a Day; numbers 1–6.

### Testing procedures

Baseline testing was completed over a three-week period ending at the conclusion of the first week of the Walk Kansas program, and prior to the receipt of the initial program newsletter. Participants who volunteered for the study came to a university fitness laboratory to complete their questionnaires. Before filling out their surveys, participants were given an informed consent sheet to read and sign, giving permission to use their data.

### Instruments

#### Physical activity

The BRFSS physical activity items were revised for a written format and completed with the Walk Kansas registration form. Participants' level of physical activity was determined from this measure [23]. This survey determines the frequency and duration of physical activity participation at either moderate or vigorous intensity. This questionnaire has been previously used to determine behavior characteristics in the health arena [31,32]. Reliability has been found to be above .70 for behavioral risk factors [33].

#### Fruit & vegetable consumption

The National Cancer Institute Fruit & Vegetable Screener was used to determine F&V consumption. This 10-item scale determined if a specific food was consumed, how frequently it was consumed, and how large each serving size was. From these data it was possible to determine the total servings per day an individual consumed. This measure has been used and was found to be reliable and valid in several studies [34-36].

#### Self-Efficacy and outcome expectations

SE for F&V consumption was measured with seven questions, answered on a five-point Likert scale ranging from not at all confident to completely confident. The questions were worded to ask the level of confidence the subject possessed for their ability to make F&V available, structure their meals to include F&V, replace current snacks with F&V. Confidence in their ability to make good F&V choices, to prepare F&V so they are nutritional and appetizing, and their ability to select good tasting F&V, were also assessed. OE regarding the benefits of consuming F&V were assessed with four questions evaluated on a five-point Likert scale, ranging from not at all likely to extremely likely. Questions evaluated participants' thoughts on the likelihood of whether or not F&V consumption would reduce their risk of cancer, improve overall health, reduce risk of heart disease, and help maintain or lose weight. Both scales had adequate internal consistencies (α >.75).

### Statistical analysis

Baseline characteristics of the study participants were analyzed through descriptive statistics including means and percentages. A multivariate analysis of variance (MANOVA) was used to compare baseline data between participants who were randomly assigned to each condition. Repeated-measures ANOVAS were conducted to determine the within and between subjects effect of the intervention on social cognitions and F&V. Tests of mediation were conducted using the method outlined by Baron & Kenny [37].

## Results

### Demographics

The participants were primarily female (75%), Caucasian (83%), well-educated (87.5% with more than a high school education), and employed full time (75%). Chi squared analysis on these categorical variables by condition of random assignment showed no differences between the control and intervention participants. The mean age of the sample was 41.4 (SD = 13.1) years, however the control condition participants (37.0 ± 11.7 years) were significantly younger than those randomly assigned to the experimental condition (44.6 ± 13.6 years; F(1,84) = 7.6, p < .01). At baseline participants reported consuming on average 5.5 ± 3.3 servings per day. Baseline values were virtually identical between control and intervention conditions. As age could reflect a potential confounding variable we completed regression analyses that demonstrated that age was not related to F&V at baseline, follow-up, or to the change in F&V over time. Two participants were excluded from analyses because their daily reporting of F&V exceeded realistic ranges (i.e., 34 servings per day) leaving a sample size at baseline of 86 participants.

### Representativeness of sample

The population of interest was adults between the ages of 18 and 65 who were enrolled in a physical activity promotion program. When compared to the total population (n = 586), the sample in this study is quite representative. The average age of the population was 42.4 years (± 13.98) whereas the sample of study participants was 41.4 years (± 13.1). Almost 90% of the population was Caucasian and 75.6% female which was comparable to the sample (83% Caucasian, 75% female). Within the population, 55.3% were meeting the minimum recommendations for physical activity. Based on the inclusion criteria for this study, none of the participants were at that level of physical activity at the beginning of the intervention. No significant differences were found between individuals who agreed to participate in the study and those who declined.

### Attrition rate

Thirty percent of the baseline sample was lost to attrition (n = 26). Chi squared analyses showed no significant differences in attrition between intervention (33%) and controls (27%). Several individuals were scheduled for their post-test data collection, yet failed to attend their appointment (n = 7). Several subjects were unavailable through telephone communication to schedule their appointments (n = 15). Other subjects included in this attrition number are those who did not provide complete data sets (n = 4). Chi squared analyses also revealed no significant differences in demographic characteristics between those who returned for post-test and those who dropped out. However, those who dropped out of the intervention consumed more servings of F&V per day, on average, (6.5 ± 4.0) than those who remained in the study (5.0 ± 2.0 [F(1,84) = 3.9, p = .05]).

### Primary analyses

Both an intention to treat analysis, where baseline scores were used as follow-up scores for participants lost to attrition, and an actual treatment analysis, using only those participants who completed the intervention and follow-up assessments were completed. In each case, repeated measures analyses of variance (ANOVA) using time as the within subject independent variable and condition as the between subject independent variable was used. Repeated measures ANOVAs were completed with total daily servings of F&V, daily servings of fruits, and daily servings of vegetables as the dependent variables.

#### Intention to treat analyses

For this set of analyses, a conservative approach was used to account for missing data. Participants' baseline scores for F&V consumption were carried forward and used as follow-up scores, indicating no change regardless of condition assignment.

The initial repeated measures ANOVA examined changes in total F&V consumption over time and by condition of randomization. A significant time by condition interaction (F(1,84) = 4.67, p < .05) indicated that participants who received the SCT intervention increased by approximately one serving of F&V per day whereas control participants did not increase number of servings per day (Table 2).

**Table 2:** Intention to treat analyses: Changes in fruit and vegetable consumption by condition.

	Intervention		Control		Sig.
	Baseline	Follow-up	Baseline	Follow-up	
F&V	5.5 (.50)	6.4 (.49)	5.4 (.52)	5.3 (.52)	p < .05
Fruits	1.9 (.30)	2.4 (.33)	2.2 (.33)	2.2 (.35)	p > .05
Vegetables	4.6 (.54)	4.9 (.52)	3.2 (.58)	3.1 (.56)	p > .05

To determine if the differences between conditions were due to increases in both fruit and vegetable consumption or changes in either fruit or vegetable consumption alone, repeated measures ANOVAs examined changes in fruit and then vegetable consumption over time and by condition of randomization. When analyses were completed for fruits and vegetables in isolation of one another the time by condition interaction was not significant for consumption of fruits (F(1,84) = 3.56, p >. 05) or vegetables (F(1,84) = 1.51, p > .05) potentially indicating that changes found in combination were the result of modest changes in consumption of both fruits and vegetables (Table 2).

#### Present at follow-up analyses

A significant time by condition interaction (F(1,58) = 5.24, p < .05) indicated that participants who received the SCT intervention increased by approximately 1 and 1/3 servings of F&V per day whereas control participants did not increase number of servings per day (Table 3). Similar to the intention to treat analyses we determined if the differences between conditions were due to increases in both fruit and vegetable consumption or changes in either fruit or vegetable consumption alone using repeated measures ANOVAs. A significant time by condition interaction for consumption of fruits (F(1,58) = 4.24, p < .05) indicated that participants who received the SCT intervention increased by approximately .75 of a serving of fruits per day whereas control participants did not increase number of servings per day (Table 3). The time by condition interaction was not significant for consumption of vegetables(F(1,58) = 1.61, p > .05).

**Table 3:** Present at follow-up analyses: Changes in fruit and vegetable consumption and social cognition by condition.

	Intervention		Control		Sig.
	Baseline	Follow-up	Baseline	Follow-up	
F&V	4.8 (.55)	6.1 (.55)	5.2 (.54)	5.1 (.55)	p < .05
Fruits	1.5 (.27)	2.3 (.33)	1.9 (.27)	2.0 (.33)	p < .05
Vegetables	3.3 (.42)	3.9 (.39)	3.3 (.42)	3.2 (.39)	p > .05
Self-Efficacy	3.4 (.14)	3.5 (.15)	3.4 (.14)	3.6 (.15)	p > .05
Outcome Likelihood	4.0 (.13)	4.3 (.12)	4.0 (.13)	4.3 (.12)	p > .05
Outcome Value	4.4 (.11)	4.4 (.11)	4.4 (.11)	4.4 (.11)	p > .05

### Examination of potential mediators

The SCT intervention was developed to target increases in SE and OE related to F&V consumption that would mediate changes in consumption. The first condition to satisfy mediation as outlined by Baron & Kenny [37] is that an intervention has an effect on the primary outcome. This criterion is satisfied by the analyses described above. The second criterion necessary is that the intervention has an effect on the proposed mediators, SE and OE. To determine if this criterion was met we completed a repeated measures ANOVA on each of our proposed mediators. To enhance the variability of responses and maximize the likelihood that participants received the intervention, these ANOVAs were completed with only those participants who were present at follow-up. Participant perceptions of SE (F(1,58)=.02, p > .05) and OE (F(1,58)=.002, p > .05) did not differ by condition, time of assessment, or condition by time of assessment (Table 3). As a result we conclude that the targeted variables did not mediate the effect of the intervention.

### Intervention implementation

In order to test the level of receipt of the intervention and its influence on effectiveness of the intervention, the subjects were asked about the number of newsletters they received and how many they read. The mean number received was 7.47 (+/- 1.57), and the mean number read was 6.71 (+/-2.27). These numbers were comparable between the conditions.

Although the hypothesized mediation relationships were not found, the dose of intervention may have impacted effectiveness. That is, those participants who read more newsletters should increase their F&V consumption to a great extent when compared to those who read fewer newsletters. Participants in the FVI condition were dichotomized into two groups based on the mean number of newsletters read. Given the small sample size an a priori .10 level of significance was used to detect differences in changes in total F&V consumption. Those participants who read seven or more newsletters increased their number of servings of F&V by two per day (1.99, SE = .47) whereas those who read fewer had virtually no change in consumption (0.02 servings, SE = .40; F(1,28) = 3.67, p < .10).

## Discussion

The findings of the study can lead to a number of generalizations. First, the study population consumed, on average, approximately the minimum number of servings of F&V at baseline but did not approach the upper limit of the recommended levels (i.e., nine servings per day). Second, the intervention successfully, although modestly, increased self-reported F&V consumption. Third, participants who reported reading seven or more newsletters had a more robust effect from the intervention. Fourth, changes in social cognition did not mediate the relationship between the intervention and F&V consumption. Fifth, utilizing a program targeting a single health behaviour is an effective way to address changes in an additional health behaviour. Other noteworthy findings include the impact of attrition on study findings and the potential for dissemination of the intervention.

At baseline, the sample in this study consumed approximately the minimum number of F&V servings per day as recommended in Healthy People 2010 [38]. This finding is similar to that found by Krebs-Smith and Scott Kantor [10]. Their data suggest that American adults (age >/= 20 years) are self-reporting consumption on average of 5.2 servings of F&V per day. When they divided the country by region, they found that adults in the Midwest were consuming an average of 5.0 servings of F&V per day. Also found in this study was the propensity for Americans to be increasing their consumption of F&V over time. The authors stated that there is a growing proportion of elderly, nonsmokers, and people with higher education and/or income levels within the population and that these groups tend to consume more F&V than others [10]. Our study sample was primarily middle class, Caucasian females who may also consume more F&V than the general population. This speculation is supported by the study done by Mayer et al. [39] which found that females ate more F&V than males, whites more than blacks, and older age groups more than young adults.

The subjects in the FVI group did increase their F&V consumption during the course of this intervention over that of the control condition. When excluding the data from drop-outs, there is a significant increase in F&V consumption by 1 1/3 servings. These results are similar to those seen in other studies. Marcus et al. [40] conducted an intervention to increase F&V consumption on callers to the Cancer Information Service (CIS). Those individuals who received the intervention, which consisted of a proactive educational message given over the phone along with two follow-up mailings, significantly increased their F&V consumption by .88 servings a day compared with the control condition. In a study involving a computer-assisted diet intervention, the adjusted mean difference between control and intervention conditions for F&V consumption post-intervention was .93 servings per day [41]. Steptoe et al. [29,30] found a significant increase in F&V consumption (1.49 servings/day) when using a theory-based behavioural counselling technique with low-income adults.

In contrast to this previous research, our study used newsletter messages in conjunction with a physical activity promotion program whereas the other studies were focused on diet alone and included more intensive programs. Intervention models that target a single behaviour, as in the studies detailed above, have been criticized as inefficient and impractical [14]. By integrating F&V messages into an ongoing program that promotes regular physical activity, low fat eating, and healthy portion sizes, the behavioural impact of this program is being optimized. This finding is also consistent with previous research using print materials. For example, Baranowski et al. [42] found that the delivery of age-specific print materials to Boy Scouts and their parents was effective in increasing F&V consumption in these children. Lutz et al. [43] found that delivery of newsletters regarding F&V consumption was successful at increasing F&V consumption in adults and that the general messages were just as effective as the computer-tailored ones. Combined with the findings of the present study, the research literature suggests F&V messages that are theory-based, but non-tailored, may still bring about a significant increase in F&V consumption, similar to that seen in other more intensive interventions [29,40,41].

There is some question about the cost and practically of many behavioural interventions developed in environments that are static and do not address issues of external validity [44]. We proposed to test nine, 500-word messages delivered in an existing program, thereby leveraging the funds and labour hours necessary to deliver one intervention to deliver a second intervention at no additional cost to the system. As there was no additional cost to implement this intervention, it has the ability to be integrated into existing programs and to be delivered by additional organizations, thereby increasing its public health yield.

Contrary to our hypothesis, there were no observable mediation effects between FVI, SE, OE and F&V consumption. This finding may lead one to question the utility of social cognitive theory as the basis for intervention effectiveness and thereby for intervention development. However, the entire content of this intervention was developed using Bandura's [26] theory – and the intervention was effective. One possible explanation for the lack of mediation is that the measures used to assess SE and OE were not sensitive to change (also note the potential ceiling effect with the outcome expectation variables). Further, given the numerous forms of self-efficacy and outcomes of eating F&V it is possible that the measures developed for this study, were not tapping the appropriate dimensions of these social cognitive variables. Finally, it could also be that this print form of intervention does not influence self-efficacy and outcome expectations per se, but may affect the actual skill level necessary for participants to improve their eating habits.

This study has demonstrated that a low-cost F&V newsletter intervention is effective when delivered in combination with a community-based physical activity promotion program. As such, this intervention has several characteristics that give it high potential for translation into practice. Low cost, ability for broad reach, and effectiveness at health behaviour change are all components of a successful translational program. However, there were some limitations associated with this trial. First, F&V consumption was determined through self-report recall measures. Participants in FVI may have read their newsletters and felt the emphasis being placed on F&V intake, and decided to report eating more F&V without actually following through with their behaviour. Second, as subjects were also participants in a pay for use physical activity promotion program, they could have been more motivated to change health behaviors than the general population [45]. Studying individuals who have such an elevated level of motivation could create results that are not generalizable to a broader community population. Related, although social economic status of the participants was not assessed in this trial, there is typically differential uptake across socio-economic strata and this method of intervention should be tested across a variety of contexts to demonstrate effectiveness and applicability for those at highest risk.

## Conclusion

This brief, inexpensive F&V newsletter intervention was as effective for increasing F&V consumption in adults as other, more labour intensive, F&V interventions. Minimal labour cost interventions using newsletter delivery have the potential to reach large proportions of society at one time. Thus, this type of program has excellent potential for public health yield.

## Competing interests

The author(s) declare that they have no competing interests.

## Authors' contributions

SD participated in study coordination, data acquisition, intervention design and drafted the manuscript.

PE conceived of the study, led intervention development, performed data analyses and assisted in drafting the manuscript.

Both authors have read and approved the final draft of the manuscript.
